# Book Review

**Published:** 1943

**Authors:** 


### Reviews of Books

**Treves' "Students' Handbook of Surgical Operations."** By C. P. G. Wakeley, C.B., F.R.C.S. Seventh Edition. Pp. xii., 562; 265 illustrations. London: Cassell & Co. Ltd. 1943. Price 12s. 6d.—This hardy perennial first made its appearance in 1892 and there must be few medical men who do not remember from student days the excellent plates illustrating amputations and ligation of vessels. Many of them have successfully weathered the half-century. Even such set operations have been drastically pruned in accordance with modern practice and a large part of the volume has been completely re-written. One might criticize some of the operations, *e.g.* phrenic avulsion as a treatment for bronchiectasis; excision of the head of the first metatarsal for hallux valgus; disarticulation through the wrist joint (which makes the fitting of an artificial hand very unsatisfactory); and the introduction of a Smith Petersen pin only by means of an open operation. Curiously enough, excision of the condyle of the mandible is described twice: once on page 198, with an illustration in which the temporal muscle is labelled the masseter, and again on page 245. In spite of these few small blemishes a student or house surgeon will find this handy little book packed full of useful and easily assimilable information.

**The Medical Annual, 1943.** Edited by Sir Henry Tidy, K.C.B., and A. Rendle Short, M.D., F.R.C.S. Pp. lx., 432, 75. Bristol: John Wright & Sons, Ltd. Price 25s. net.—Although the *Medical Annual* for 1943 is somewhat late in coming out we are glad to find that the Ministry of Supply has allocated sufficient paper to allow of its being slightly larger than the former issues during this war. This recognition of its great value to the Medical profession is well merited. Wartime medicine and surgery are continually changing and progressive, thus Wright's *Medical Annual* is even more indispensable now that current literature, except that published at home, is hard to come by. Perhaps the most important feature of this year's *Annual* is Mr. D. Harcourt Kitchin's summary of "Legal Decisions" affecting the medical profession, especially that which concerns the liability of hospital authorities for negligence of their medical officers. Wakeley on "Burns of War" is first rate. The articles on "Typhus" and "Yellow Fevers" are of great importance to all service M.O.'s. In the introduction special attention is directed to articles dealing with the "Dangers of New Therapeutic Measures," three particular groups of treatment are singled out for warning namely, sulphonamide administration, electro-convulsive therapy and the management of Addison's disease. In every branch of the Art and Science of Medicine practitioners will be safer and wiser for reading Wright's *Annual*.

**Tropical Nursing.** Second Edition. By A. L. GREGG, M.A., M.D., M.Ch., D.T.M. & H. 1943. London: Cassell & Co. Ltd. Pp. x., 185. Price 6s. net.—Dr. Gregg's little book can be warmly recommended to nurses and ordinary folk going to the tropics. Thanks to the care with which the first edition was prepared, few changes in the advice on nursing have been needed in the second edition. Such changes as there are have occurred mainly in treatment, that is to say, drugs and diet. The text is clearly written, and if treatment must, in emergency, be given before medical aid can be obtained, this book should be of real help. On one or two points of treatment, criticisms suggest themselves. On page 92, quinine *per rectum* is advised in certain circumstances, but this method has been abandoned by the majority of authorities as inefficacious. Even if ampoules are not available it should be possible for a nurse to boil a solution of quinine in a test-tube for intra-muscular use and render it sterile. Antimony has proved of great value in a number of tropical diseases, and considerable progress has been made in developing new compounds. When, therefore, it is pointed out that for the treatment of bilharziosis other antimony compounds are superseding the tartrate, it was a pity not to mention such compounds as has been done in the case of kala-azar (stibosan, stibamine and diamidinostilbene). For ulcerating granuloma antimony tartrate is still (p. 140) the only remedy mentioned, although neo.-stibosan is now usually preferred. Under treatment of ascaris (p. 69) some excellent advice is given about the use of vermifuges, and the safety of employing castor oil with santonin is wisely pointed out. The dangers of oil of chenopodium for very young children and of repeating the dose of carbontetrachloride are, unfortunately, not mentioned. Deeks never gave oil of chenopodium to children under four years of age, and always followed it with a purge of salts within one to one-and-a-half hours. The comparative safety of hexylresorcinol is rightly mentioned. Dr. Gregg makes an interesting recommendation (p. 6) that dark-coloured or black underwear should be worn in the tropics. Has dark underwear been proved in practice to be cooler? There are possible disadvantages in dark-coloured underwear unless high quality fast dye is used. Perspiration might cause an inferior dye to run, with resultant irritation of the skin and possible sepsis. The book as a whole is so good that it is hoped that the author may consider these few points of criticism when he comes to prepare a new edition—perhaps, too, he will see that Sir Philip Manson Bahr's name is correctly spelt on p. viii.

**Sixth Addendum to British Pharmacopoeia. 1932.** Official from August 1st, 1943. Six months' grace allowed for B.P. Ointments amended in this Addendum. The Addendum contains a number of very important additions and alterations.

ADDITIONS.

ACETOMENAPHTHONUM. MENAPHTHONUM. Oral and parenteral forms respectively of Vitamin K., hitherto chiefly prescribed and supplied as the products of various firms under their own Brand names.

RIBOFLAVINE. The new official form of Vitamin $B_2$, recognizing both natural and synthetic sources.

AC. RICINOLEIC TERPINEOL. Both included for use in the preparation of Liq. Chloroxylenolis.

LIQ. CHLOROXYLENOLIS. A useful antiseptic of the Dettol type.

DITHRANOL. A yellow powder for use in skin ointments and pastes, replacing a well known German proprietary.

NICOTINAMIDE. A more modern application of the Pellagra Preventive Vitamin.

STILBOESTROL. Synthetic Oestrogenic substance for oral and parenteral use. The Addendum defines it further as DIETHYLSTILBOESTROL.

INSULIN. Storage conditions and pH. limits extend the period of potency to two years from date of manufacture.

ERGOT. The monograph now takes Ergometrine as the basis of activity instead of Ergotoxine.

SYR FERRI PHOSPH. c STRYCH. The equivalent of Easton's Syrup without the Quinine, which is now not available except for Malaria treatment.

SULPHANILAMIDE. The monograph on its sterilization establishes 150° for one hour, or 140° for four hours, as the heat limits for efficient sterility.

ALCOHOLIA LANAE. The product of saponification of the grease of sheep's wool with fractionation of part containing Cholesterol (28 per cent.), and other Alcohols is introduced as the basis for preparing :—

1. Ung. Alcoholia Lanae.

2. Ung. Aquosum.

3. Ung. Ac. Boric.

4. Ung. Ac. Sal.

5. Ung. Ac. Tannic.

6. Ung. Hamamelis.

7. Ung. Hyd. Ammon.

8. Ung. Hyd. Oleat.

9. Ung. Hyd. Subchlor.

10. Ung. Hyd. Sulph.

11. Ung. Zinc. Ox.

This is a notable alteration in B.P. Ointment bases, and it remains to be seen whether the skin physicians will approve the change. The new ointments are of very nice smooth consistence and appear to be an improvement on the older ointments.

KAOLIN. Now appears in two forms, Light and Heavy ; the former for internal use and the latter for the manufacture of Cataplasma Kaolin Co.

MAG. TRISILICATE. New standards for Silicon content are laid down.

INJECTIO PROCAINE AND ADRENALIN. The title of this preparation which contained 2 per cent. of Procaine is changed to INJECTIO PROCAINE AND ADRENALIN FORT, and a weaker preparation is introduced under the name of INJECTIO PROCAINE AND ADRENALIN MITIS, containing only about ½ per cent. of Procaine.

**Diseases and the Social System.** By ARTHUR GUIRDHAM, M.A., D.M., B.Sc., D.P.M. Pp. xvi., 239. London: George Allen and Unwin Ltd. 1942. Price 10s. 6d.—" This book is written for the profession in general, and in particular for the inarticulate majority, for whom man and his sufferings are of greater moment than the findings of group research." It is regretted that a reviewer has not been found from the inarticulate majority, but in order to bring the book to its notice some description of its contents will be given. From the dust-cover one learns that the book emphasizes that disease is due largely to our social system, it argues that the beliefs we inherit are responsible not only for psychological but for physical disease. Most disease, the author holds, is a response to, or an evasion of, strain. At present the administration of Society rests largely in the hands of the abnormal and diseased. The author deplores the influence of Pasteur, saying " he retarded the progress of medicine by a hundred years." He feels that some day psychiatry and general medicine will have merged, at present psychiatry is a branch of medicine. The position should be reversed. But not until psychiatry asserts itself in the domain of physical disease. Surgery he dislikes as being blind carpentry performed by persons of inadequate culture. When, despite medical treatment, the violent corrective of surgery must be applied, " we are making a patchwork effort for those who have failed to adjust to circumstances as a whole." The maimed victim of a high explosive bomb can scarcely be blamed for maladjustment to circumstances, but let that pass. The author divides his book into four sections. The first deals with personality in relation to disease. The second explains how most chronic disease, physical as well as nervous, is neuropathic in nature and attributable to the effect of abnormal emotion on vulnerable personalities. The third reviews the effect of the social system in providing a background conducive to abnormal emotion. The last section deals with tendencies in medicine which prevent our dealing adequately with current problems. At the end of the book the author's chief reason for writing it is manifest. " We doctors," he writes, " must insist on playing a major part in the cure of a sick world." Well, yes, perhaps we doctors might, provided we are as humble as Paré : " *Je le pansay, Dieu le guarit.*" But I doubt whether the author intends to play the part of a bandager, waiting for the divine healer. It is interesting to see the influence upon a psychiatrist of observing psychical causes produce organic changes in the human body.

**Outlines of Industrial Medicine, Legislation and Hygiene.** By JAMES BURNET, M.D. Pp. 87. Bristol : John Wright & Sons Ltd. Price 7s. 6d.—This small and readable volume is intended for students and others who may require an introduction to the subjects with which it deals. Part I is devoted to Industrial Diseases, and includes Poisons, Medical Diseases, Surgical Affections, Diseases of the Eye, and Skin Diseases. The descriptions are concise and on the whole accurate. Part II discusses Industrial Legislation. The author is not always reliable in his accounts of the Law, although his statements may accurately describe things that are customary, although not lawful. For instance. In his chapter (V) on Skin Diseases, he says that if a workman's application to an appointed surgeon for a certificate that he is suffering from a compensatable disease is refused, he will get neither National Health Insurance benefit nor compensation. But as the law stands the workman who is refused a certificate under the Workmen's Compensation Acts is entitled to N.H.I. benefit, provided he has his panel doctor's certificate that he was unfit for work. The decision of the certifying surgeon, not that of the Approved Society, is final, unless disturbed by the Medical Referee on appeal. The author does not at this point mention that a refusal of a certificate by the appointed or certifying surgeon that a disease or injury comes under the Workmen's Compensation Act can be appealed against by the workman. A little further on (p. 50) he says that because a skin condition may have cleared up, the Referee on appeal " can only certify that the worker is not suffering from dermatitis." This is frankly nonsense. The Referee is bound to accept the " appointed " surgeon's certificate unless evidence is brought to show that when the workman was examined " by the examining surgeon " he was not, in fact, suffering from the scheduled disease. The certificate is sufficient and final evidence that the workman was so suffering until upset by convincing evidence to the contrary. That the workman is not so suffering at the time of his examination by the Medical Referee should not, without other evidence, cause the Referee to allow an appeal. A grave omission on the author's part is his failure to quote the obligation placed on Approved Societies by the National Health Insurance to pay a workman weekly benefit " as an advance by way of a loan " where the workman has applied for compensation and the employer disputes his liability to pay or delays payment. Nowhere does the author remind his readers that the certificate of the certifying surgeon must state that the dermatitis is " produced by dust or liquids." He is content to say " practically anything to which a worker's skin may become senstitized will readily produce dermatitis." He does not add that the schedule to the Acts only recognizes dermatitis produced by dust or liquids. Doubtless the Acts need amendment, but there is the law as it stands. The book is a good book as a guide to Industrial Diseases, but, on questions of Law, Willis or Butterworth is greatly to be preferred. And it is most desirable that industrial surgeons, as well as certifying surgeons and medical referees, should know the legal as well as the medical aspects of the work they undertake. The author gives the impression that he has not studied Case Law on his subject.

